# Migration of Chemical Compounds from Packaging Materials into Packaged Foods: Interaction, Mechanism, Assessment, and Regulations

**DOI:** 10.3390/foods13193125

**Published:** 2024-09-30

**Authors:** Rakesh Kumar Gupta, Sunil Pipliya, Sangeetha Karunanithi, Gnana Moorthy Eswaran U, Sitesh Kumar, Shubham Mandliya, Prem Prakash Srivastav, Tejas Suthar, Ayaz Mukarram Shaikh, Endre Harsányi, Béla Kovács

**Affiliations:** 1 Agricultural and Food Engineering Department, Indian Institute of Technology Kharagpur, Kharagpur 721302, India; rk86214@kgpian.iitkgp.ac.in (R.K.G.); sunilpipliya22@gmail.com (S.P.); sang0306@kgpian.iitkgp.ac.in (S.K.); gmeswaran@kgpian.iitkgp.ac.in (G.M.E.U.); shubham.mandliya15@gmail.com (S.M.); pps@agfe.iitkgp.ac.in (P.P.S.); 2 Independent Researcher, Chicago, IL 60616, USA; suthartejas2525@gmail.com; 3 Faculty of Agriculture, Food Science and Environmental Management, Institute of Food Science, University of Debrecen, 4032 Debrecen, Hungary; ayaz.shaikh@agr.unideb.hu; 4 Doctoral School of Food Science & Nutrition, University of Debrecen, Böszörményi út 138, 4032 Debrecen, Hungary; 5 World Food Forum, I-00100 Rome, Italy; 6 Agricultural Research Institutes and Academic Farming (AKIT), Faculty of Agriculture, Food Science and Environmental Management, University of Debrecen, 4032 Debrecen, Hungary; harsanyie@agr.unideb.hu

**Keywords:** migration mechanism, migrating compounds, chromatographic techniques, regulations/legislations

## Abstract

The migration of chemical compounds from packaging polymers to food presents a multifaceted challenge with implications for food safety and public health. This review explores the interaction between packaging materials and food products, focusing on permeation, migration, and sorption processes. The different migration mechanisms of contact migration, gas phase migration, penetration migration, set-off migration, and condensation/distillation migration have been discussed comprehensively. The major migrating compounds are plasticizers, nanoparticles, antioxidants, light stabilizers, thermal stabilizers, monomers, oligomers, printing inks, and adhesives, posing potential health risks due to their association with endocrine disruption and carcinogenic effects. Advanced analytical methods help in the monitoring of migrated compounds, facilitating compliance with regulatory standards. Regulatory agencies enforce guidelines to limit migration, prompting the development of barrier coatings and safer packaging alternatives. Furthermore, there is a need to decipher the migration mechanism for mitigating it along with advancements in analytical techniques for monitoring the migration of compounds.

## 1. Introduction

Public health and worldwide food safety are gravely threatened by the migration of chemical compounds from packaging polymers into food systems. As food systems continue to evolve, the interaction between food products and packaging materials has become increasingly complex, with the potential for additives and contaminants to leach into food matrices during storage, transportation, and even preparation [1,2,3]. The interactions between food and packaging material are driven by permeation, migration, and sorption. Migration denotes the transference of chemical components, including additives, plasticizers, contaminants, or other chemicals, from packaging materials into food products. This process may affect both food safety and quality, since some compounds may present health hazards (e.g., endocrine disruptors and carcinogens), while others, like antioxidants or vitamins, might provide advantageous benefits by preserving food and prolonging shelf life [4,5,6]. There are two types of migration: global and specific. Global migration is the aggregate amount of all chemicals that migrate from the packaging materials to food, usually calculated as the total of all migrated compounds without differentiating particular components. Specific migration pertains to the migration of distinct, recognizable chemical compounds, such as plasticizers or additives, from packaging materials into food, often subject to specific limitations for each component. The packaging material must be subjected to a specific time-temperature condition in order to ascertain the quantity of the migrating compound [7]. Due to the complexity and variability of testing with real food under real environments, migration testing is generally conducted using food simulants. Simulants replicate the characteristics of various food types, allowing for the regulated and standardized measurement of the migration of chemicals from packaging materials [8]. A food simulant is a standardized compound that imitates the characteristics of different food groups, such as moist, fatty, or acidic foods [9].

The migration phenomenon, driven by mechanisms such as diffusion, partitioning, and permeation, involves a wide range of substances, including plasticizers, antioxidants, and stabilizers, which are incorporated into packaging materials to enhance their performance and durability [9,10]. There are numerous variables that can impact the interaction between food and packaging material, such as the chemical properties of the packaging materials, the composition of the food, the temperature, and the duration of contact between the packaging and food [2]. For instance, fatty or acidic foods tend to promote the movement of certain compounds, like bisphenol A (BPA) and phthalates, which have been linked to negative health consequences [11]. Furthermore, the oxidative reactions, and the loss of flavor and nutritional value, can be exacerbated by the permeation of gases, moisture, and volatile compounds from packaging, which can further degrade food quality [12]. In order to guarantee food safety and the advancement of safer packaging materials, it is imperative to conduct a thorough investigation of these intricate interactions.

To address these issues, regulatory bodies, such as the U.S. Food and Drug Administration (FDA) and the European Food Safety Authority (EFSA), have implemented standards to restrict the transfer of hazardous chemicals from the packaging to food (Regulation (EC) No. 1935/2004) [13]. These rules mandate the strict testing of packaging materials and establish migration limits for harmful compounds like BPA and phthalates in an effort to safeguard consumers. Based on the outcomes of these risk evaluations, specific migration limits (SMLs) and/or further limitations have been imposed on some drugs. For all other substances on the Union list, the overall migration limit (OML) of 60 mg/kg of food is applicable. Conversely, there are no EU-wide standardized standards for non-plastic food contact materials (FCMs) such as paper and board, metals and alloys, silicones, and rubbers. Manufacturers of these products should adhere to national regulations, if available, or conduct risk assessments to be provided to authorities upon request. They may further consult the ESCO list [14], which was begun by the European Food Safety Authority (EFSA) and created by EFSA’s Scientific Co-operation Working Group (ESCO). The list has around 3000 items of chemicals used in non-plastic food contact materials. This list offers information at the Member State level, together with industry expertise [14]. The European Chemicals Regulation (Regulation (EC) No. 1907/2006) concerning the Registration, Evaluation, Authorization, and Restriction of Chemicals (REACH) [15] seeks to safeguard human health and the environment against chemical hazards.

Sophisticated analytical methods, including chromatography and mass spectrometry, have facilitated the more accurate identification and measurement of migrating chemicals, therefore assisting in adherence to regulatory requirements [16,17,18,19,20]. These methodologies have been shown to be crucial in the detection of minute quantities of toxic compounds in food, thereby assisting in the reduction of hazards linked to chemical migration [18,19,20]. Nevertheless, there are still substantial gaps in understanding, especially about the long-term health consequences of prolonged exposure to low concentrations of migrating chemicals.

In this context, this study aims to critically examine the challenges that arise from the migration of chemicals from packaging materials to food systems. By exploring the interaction between food and packaging materials, this study investigates the mechanisms that drive the migration of chemicals/additives from the packaging material into the food. It also identifies the migrating compounds, assesses the potential toxicity associated with these compounds using the analytical techniques and evaluates the regulation to mitigate its risk. By addressing the complexities of chemical migration in food packaging, we can work towards building a safer and more resilient global food system for present and future generations.

## 2. Interaction between Packaging Material and Food

**Figure 1:**
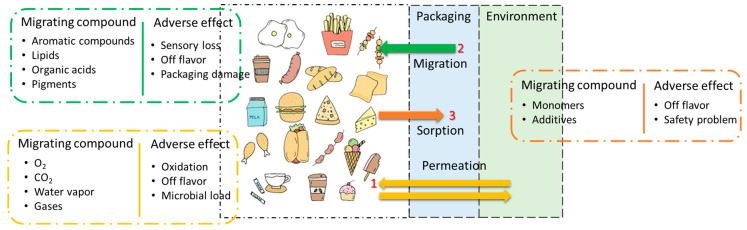
Interaction between packaging material and food.

The manner in which food products and packaging materials interact significantly influences the quality, safety, and longevity of the food [21]. This interaction involves complex chemical, physical, and biological phenomena that necessitate careful examination. It includes mass transfer where the compounds are migrated from the packaging material to the food either by permeation, migration, or sorption phenomena individually or as a combination of them simultaneously (Figure 1) [21,22]. This mass transfer is influenced by Fick’s and Henry’s law of diffusion, which dictates substance movement through materials based on concentration gradients.

**Figure 2:**
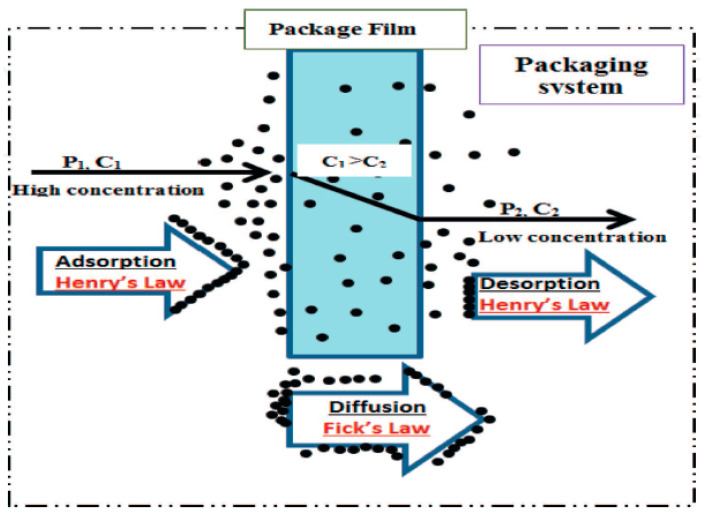
Principle of permeation in food packaging (adapted from [21]). (Dots as gas or vapor molecules; arrows are showing the direction of flow that is from higher to lower concentration; the black color word is the mechanism and the red color word is the law).

Permeation is a crucial phenomenon in the realm of food packaging, involving the movement of gases (such as carbon dioxide and oxygen), moisture, light, aroma, flavor, and volatile compounds from packaging materials to the food or vice-versa [22]. The permeation principle is presented in Figure 2. This movement of gases may deteriorate the quality of food due to the alternation in the gas composition inside the packaging material [21]. For instance, the permeation of oxygen can lead to oxidative reactions in fat-rich food products, such as olive oil [23]. Studies have indicated that the presence of volatile terpenes can increase carbonyl formation in PET by over 60% [24].

Migration refers to the transfer of substances like plasticizers, antioxidants, or stabilizers from the packaging material to the food product. Examples include the migration of low molecular weight compounds like residual monomers, additives (antioxidants, plasticizers), and oligomers from polymeric packaging into food matrices, posing potential toxicity risks [25]. This migration of chemicals can occur at varying levels, depending on factors like the composition of the packaging material and the kind of the food product [2]. The process of migration between food and packaging materials encompasses distinct and essential stages. Initially, diffusion takes place, in which food ingredients or pollutants pass through the packaging film as a result of a concentration gradient. Subsequently, the first desorption stage involves the release of chemical substances from the packaging surface, therefore enabling their accessibility for interaction. Sorption occurs at the food-packaging contact, when components are transferred between the two surfaces by either adsorption or absorption. The second desorption process involves the migration of chemicals from the packaging into the food, which has the potential to impact the safety and quality of the food. All aspects of this process are affected by variables such as the packaging material type, storage circumstances, and the characteristics of the food.

Sorption, sometimes known as “scalping”, is the process by which packaging materials adsorb or absorb essential sensory components found in food, such as flavors, odors, lipids, and moisture [21,22]. This interaction may lower the quality of packaged food, especially in foods where flavor and odor are important. Adsorption happens when components adhere to the surface of the packaging, while absorption occurs when they permeate the material [26]. Sorption may compromise the structural integrity of packaging materials, such as low-density polyethylene (LDPE), by increasing their tendency to allow oxygen to pass through, therefore accelerating the deterioration of food [21,22,27]. The degree of sorption is dependent on the properties of the packaging material, composition of the food, and environmental factors such as temperature and humidity. Similar to migration, sorption is controlled by Fick’s law of diffusion, which elucidates the movement of molecules based on concentration gradients. For instance, studies have shown that up to 50% of d-limonene and low molecular weight aldehydes and alcohol, a key flavor compound in orange juice, can be absorbed by polyethylene-laminated cardboard packaging during a storage of 24 weeks at 4 °C [28].

**Figure 3:**
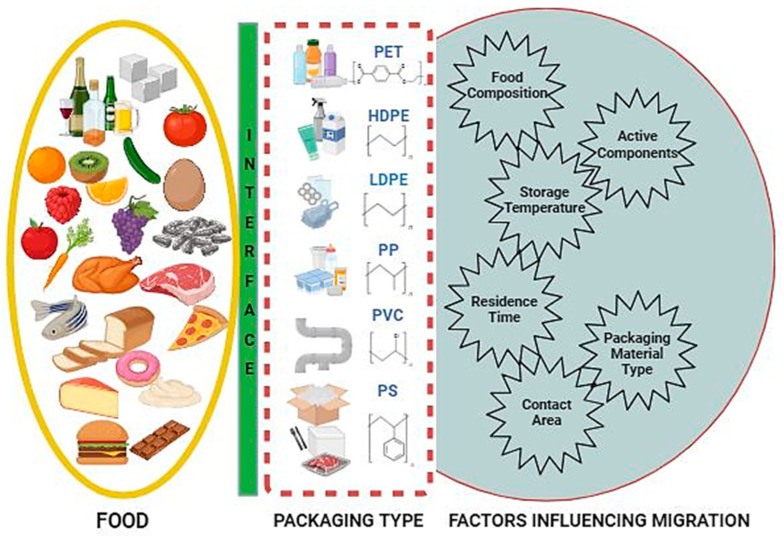
Various factors influencing the phenomenon of migration of chemical compounds from packaging materials. PET—Polyethylene Terephthalate, HDPE—High-Density Polyethylene, LDPE—Low-Density Polyethylene, PP—Polypropylene, PVC—Polyvinyl Chloride, PS—Polystyrene.

Various factors play a crucial role in influencing both permeation, migration, and sorption phenomena in food packaging (Figure 3). These factors include the composition of the packaging material, viz., the type of polymer used, additives incorporated, and the presence of coatings [29]. Moreover, food characteristics, including lipid content, moisture level, and pH, can have a substantial influence on the process of permeation, migration, and sorption [29,30]. Furthermore, storage conditions, including temperature, duration of storage, and the ratio of packaging material to food, also influence the phenomena [30]. Since these phenomena work on the principle of diffusion, the thickness of the packaging material is of the utmost importance. The thickness of the packaging material is inversely proportional to the rate of diffusion of chemicals/additives from the packaging material to the food or flavor, and aroma from the food to the packaging material [29]. Internal and external pressures or temperatures, the solubility of the components, the concentration and density of the migrating material, and other factors all influence permeability. Significant factors influencing the performance of packaging materials include their permeability, film thickness, storage duration, and relative humidity. While a lower temperature and higher thickness decrease permeation, high permeability allows gases and vapors to pass through the packaging [29]. Food physicochemical properties, storage duration and temperature, package size, and migratory movement inside the packaging material all play a role in migration [30]. Because of their higher potential for aggressive interactions with packaging materials, foods that are acidic or fatty often hasten migration. The migration rates are also considerably influenced by the type of packaging and coating used, with polymers being generally more susceptible to migration. Temperature and pressure are the primary factors influencing sorption. Sorption typically increases with increasing temperature, while there are few exceptions to this rule, including with dry casein and vinyl chloride [31]. Higher internal pressure might compromise the mechanical integrity of the packaging, thereby accelerating the rate of sorption. Furthermore, the sorption process may be expedited by the chemical composition of the sorbing substance, such as inks or surface monomers, because of their very high reactivity. Therefore, solubility is essential to determining the extent to which the volatile components are absorbed.

## 3. Mechanisms of Chemical Migration

Migration is governed by two phenomena—partition and diffusion. The partition corresponds to the migrant’s distribution between two phases (food/polymer) at the establishment of thermodynamic equilibrium. The partition coefficient (*K*) is represented as Equation (1)

(1) $$K=\frac{C_{PM}}{C_{FP}}$$

where, *C_PM_* represents migrant equilibrium concentration in the packaging material and *C_PM_* represents migrant equilibrium concentration in the food phase. When the polymer absorbs more migrants than food, *K* is greater. A high *K* restricts migration from packaging material to food, whereas a low *K* means that more migration is absorbed into the food, which is good for food safety. As a measure of the compatibility between the polymer and the solvent, the solubility coefficient influences partition coefficients, thus giving it the potential to predict the cohesive property of the packaging materials [32].

The deterministic model posits that the transfer of chemicals from packaging to food occurs via diffusion, wherein molecular structures transition from a region of higher concentration to a region of lower concentration until they attain a state of equilibrium. Diffusion in homogeneous media occurs when a migrant passes through a unit area of a section, and the rate of transfer, *R*, is directly proportional to the concentration gradient between the two sides of the package. The diffusion rate can be described using Fick’s law Equation (2).

(2) $$R=-D\left(C\right)\frac{dC}{dX}$$

where *D*(*C*) represents the diffusion coefficient in m^2^ s^−1^. To put it broadly, *D* is a function of the local diffusion concentration of *C* (mol m^−3^) and *X* shows the material thickness (m). The diffusion coefficient is an essential factor in assessing the migration potential, with the first category being minimal due to the diffusion coefficient approaching zero. The second category is invariant and is not influenced by the temporal or compositional aspects of food. The third category is directly affected by food contact, with insignificant migration occurring when there is no contact with food [33]. Aside from diffusion, there are two more processes involved in the migration stages: desorption of polymer surface molecules that have spread out, and sorption of compounds at the interface between the plastic and the food. Additionally, there is also the desorption of compounds that were previously absorbed by the food [34]. Various mechanisms of chemical migration from food to packaging material, and from packaging material to food via internal and external stimuli, are discussed below:

### 3.1. Contact Migration

**Figure 4:**
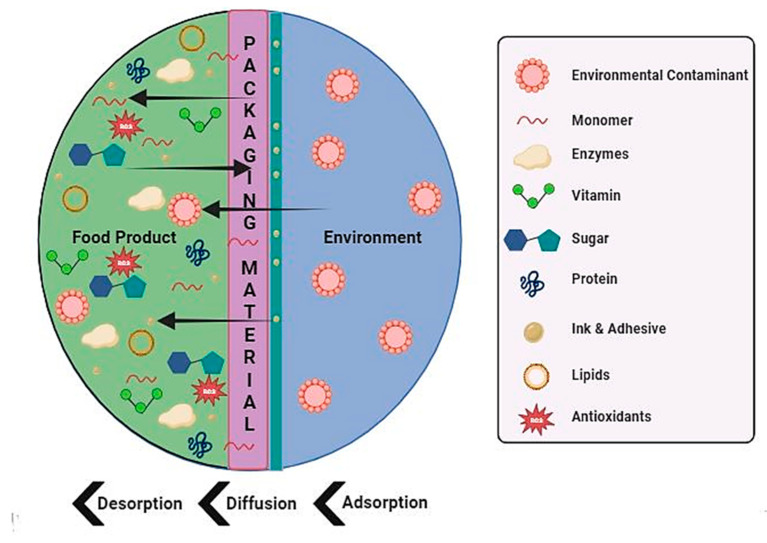
Different processes of migration of various environmental and food components.

Contact migration pertains to the transfer of chemicals from packaging materials into the food product through direct physical contact [7]. It occurs when food and packaging materials come into close contact, allowing contaminants to enter the food from the container. Diffusion is the main factor that causes contact migration (Figure 4). Molecules of substances included in the packaging material, such as additives, leftovers from manufacturing processes, or chemicals employed in package manufacture, naturally travel from locations with a greater concentration to areas with a lower concentration. When food comes into direct contact with the packaging material, substances from the container can migrate into the food [7,35]. Polymer-based packaging materials may leak substances such as monomers, additives, colorants, and contaminants upon contact with certain food [36]. Contact migration is a substantial issue in the food packaging sector since it has the potential to result in the contamination of food with hazardous or undesired chemicals.

### 3.2. Gas Phase Migration

Gas phase migration is the process of volatile chemicals transferring from packaging materials into the surrounding atmosphere and then into the food product. Contrary to contact migration, which involves direct contact between the packaging material and food, gas phase migration occurs when volatile chemicals are released and spread into the air, potentially coming in contact with the food [35,37]. The packaging materials are utilized for aromatic or flavored items, in an atmosphere that is susceptible to off-flavors, and when there is a potential presence of chemical compounds or pollutants. Volatile chemicals from mineral oil and solvents in printing inks may migrate from porous packaging materials to food via the gas phase [38].

### 3.3. Penetration (Diffusion) Migration

Penetration migration in food packaging is the phenomenon in which chemicals or impurities from the packaging material migrate into the foods by traversing the structure of the packaging. Migration of this kind takes place when the food comes into contact with the packaging, allowing contaminants such as residual chemicals, heavy metals, plasticizers, or inks to penetrate the packaging material and dissolve in the moisture, fats, or other solvents present in the food [7,35]. The extent of migration is influenced by the permeability of the packaging, the chemical composition of the packaging material, and the characteristics of the food (fatty or liquid foods have a higher capacity to absorb chemicals). Primary sources of contamination include plasticizers, heavy metals, and inks derived from recycled materials or coatings. Contaminants cause possible health hazards, including the possibility of being exposed to detrimental compounds such as endocrine disruptors, heavy metals, or toxic residues [32]. The use of barrier materials, the selection of safer packaging options, and the adherence to safety rules may effectively reduce penetration migration in food packaging [32].

### 3.4. Set-Off Migration

“Set-off migration” is the process by which ink components or labeling materials are transferred from the printed outer surface of packaging to the food product by direct contact. This phenomenon arises when printed materials, such as packaging or labels, come into contact with surfaces that are not printed, usually the side that is exposed to the food. Set-off migration occurs during the storage, transportation, or handling of packed food products. It is most prevalent when packaging materials are stacked or rolled, resulting in the transfer of ink or coating owing to physical contact or pressure [27]. In applications such as stacked packaging or vending machine cups, the ink on the printed surface may migrate to the unprinted surface, which directly touches food or drink. Under some circumstances, uncured or wet ink components may readily move via direct contact, perhaps dissolving in or contaminating the food, particularly if the food is fatty or moist, therefore facilitating the movement of soluble particles [35]. The migration process is affected by many parameters including pressure, temperature, and the chemical characteristics of the ink or coating [39]. For instance, in thermal beverage vending machines, cups constructed from plastic-coated cardboard are often adorned with printed designs on the outside and arranged in stacks. The set-off effect occurs when the ink is transferred from the outer surface of one cup to the unprinted inner surface of the subsequent cup, and then comes into contact with the hot beverage (water, milk, tea, coffee, etc.). Furthermore, the extent of set-off migration is influenced by the chemical composition of the ink or labeling materials, as well as the intrinsic characteristics of the food product [40]. The solubility of ink components in food matrices, such as hydrophilic or lipophilic compounds, might enhance their migration and possibly lead to food contamination. The existence of leftover chemicals, heavy metals, or other pollutants in packaging materials poses health hazards that may compromise the safety of the packed food [27]. Therefore, to reduce the hazards linked to set-off migration, it is essential to practise appropriate ink curing, employ safe printing materials, and implement suitable package design. These measures are necessary to avoid direct contact between ink and food, and to guarantee food safety [27].

### 3.5. Condensation/Distillation Migration

The phenomenon of condensation/distillation migration in food packaging pertains to the transfer of aromatic compounds from the packaging material to the food product via condensation followed by distillation. This process often entails the evaporation of volatile chemicals from the packaging material caused by changes in temperature or other environmental variables [7,35]. These compounds then condense on the surface of the food and subsequently evaporate again, ultimately seeping into the food. Chemical compounds that are easily vaporized, such as leftover solvents from production or volatile elements of packaging additives, might escape into the surrounding atmosphere from the packaging material [35]. When the gaseous volatile chemicals encounter colder surfaces, such as the surface of the food item or the inside surface of the package, they can undergo condensation and transform into little liquid droplets [7]. Condensation is the process by which a decrease in temperature causes vapor molecules to lose their energy and change from a gaseous state to a liquid state. The food product can absorb the condensed liquid droplets that contain volatile chemicals. Absorption takes place when the liquid droplets contact the food surface and spread throughout the food matrix by diffusion. After being incorporated into the food structure, the volatile compounds have the potential to evaporate again as a result of internal or external influences, such as elevated temperatures during storage or cooking. The volatile molecules that have evaporated might subsequently move inside the food structure, thereby impacting its taste, odor, or safety [10].

## 4. Migration of Chemicals/Additives from Food Packaging Material to Food

Food safety is profoundly concerned with the migration of chemicals/additives from packaging materials into food. Packaging materials can sometimes leach chemicals or substances into the food, which can affect the food’s safety, quality, and even taste [2,7,41]. To address this issue, food packaging materials undergo rigorous migration testing and evaluation to ensure they comply with regulatory standards regarding food contact materials. Commonly used food packaging materials such as paper, plastics, glass, and metal undergo migration testing to determine the levels of substances that may transfer from the packaging to the food. Manufacturers must ensure that their packaging materials are compliant with regulations and do not pose any health risks to consumers. There are numerous factors that affect the migration of chemicals/additives from packaging materials into food, including type of material—different packaging materials have varying degrees of susceptibility to migration), for example, plastics may leach chemicals such as BPA or phthalates, while metals may release heavy metals like lead or cadmium [42]; temperature—higher temperatures can accelerate the migration process, so food packaging must be designed to withstand the temperatures encountered during storage, transportation, and use [43,44,45,46,47]; contact time—the period of contact between the packaging and food can influence migration and prolonged contact increases the likelihood of substances leaching into the food [7]; food composition—certain types of food, such as acidic or fatty foods, are more likely to interact with packaging materials and facilitate migration [7]. To mitigate migration risks, food manufacturers may employ various strategies such as using barrier coatings, selecting appropriate packaging materials, optimizing storage conditions, and conducting migration testing to ensure compliance with regulations [1,2,41]. Therefore, the various types of migrating compounds from the packaging materials to food product are discussed in Table 1.

## 5. Types of Migrating Compounds

### 5.1. Plasticizers

Plasticizers are often utilized in food packaging as they improve the durability and flexibility of plastics [7]. Moreover, these chemical compounds are included in materials such as polyvinyl chloride (PVC) to enhance their processing and handling properties. Plasticizers can migrate from packaging materials into food, particularly when exposed to high temperatures or extended periods of storage, posing vital risks to health [7]. Plasticizers, including diethylhexyl phthalate (DEHP) and diisononyl phthalate, are phthalates that can alter the endocrine system and possibly adversely affect human health [62]. DEHP is known to disrupt the endocrine system and has been linked to reproductive toxicity, developmental issues, and cancer [62]. Phthalates are used in PVC films, plastic packaging, food container gaskets, and cap-sealing polymers [17]. Furthermore, the low molecular weight of phthalates, utilized as plasticizers for polymers, facilitates the migration of the package to the food [62]. BPA is another plasticizer often employed in polycarbonate plastics and epoxy resins, and its occurrence has been connected with a range of health concerns [62]. Various studies have reported plasticizer migration from packaging to materials to food products [46,63]. Manufacturers constantly explore alternate plasticizers or non-plastic packaging materials to mitigate migration risks.

### 5.2. Nanoparticles

Nanotechnology is a diverse and interconnected subject that focuses on using materials with sizes ranging from 1 to 100 nm [64,65,66]. Nanomaterials have a larger surface area than their larger counterparts due to their small dimensions [67]. This leads to increased reactivity and distinct features compared to the material at the macroscale level [68]. Food packaging materials increasingly incorporate nanoparticles due to their distinctive characteristics, including barrier characteristics, antibacterial activity, and mechanical strength [69]. When nanoparticles are incorporated into polymers, they serve as reinforcement. In comparison to the original polymer, their decreased permeability to gases and water results in a longer and more complicated diffusion direction, which improves barrier properties. Furthermore, the number of accessible sites in the polymer chain that are open to interactions with water molecules decreases as a result of the hydrogen bonds that form between the polymer and the nanoparticles [70]. The high aspect ratio and homogeneous dispersion of nanomaterials inside the polymer matrix are responsible for the modification of molecular mobility and relaxing of polymer chains, which leads to improved mechanical and thermal resistance [70]. Additionally, the mechanical strength of the nanocomposites can be improved by the bonding that forms between the polymer chains and the nanoparticles.

The packaging industry has the potential to integrate many nanomaterials, such as metals and their oxides (aluminum oxide, silicon oxide, titanium dioxide, copper oxide, and zinc oxide), nanocellulose, nano clays, nano silver, and essential oil nano-emulsions [69,71]. The materials most frequently employed in food packaging applications are nano silver and nano clay. Nano clay is more cost-effective than other nano-materials due to its natural origin and ecologically friendly nature [72]. Furthermore, it has been discovered that clay nanoparticles increase the mechanical, thermal, and barrier properties of polymer chains by strengthening their structural integrity through solid bonding. Strong antimicrobial qualities make copper an essential mineral. Its antibacterial properties, however, are not as potent as those of silver. Among metal nanoparticles, silver nanoparticles have the strongest antibacterial activity, efficiently combating a wide range of pathogens such as viruses, molds, and bacteria [73]. However, the utilization of silver nanoparticles is limited due to their expensive nature.

Nanoparticle migration can be facilitated by a range of processes, including diffusion, the process of desorption, and mechanical transmission [74]. Numerous elements, including the food and packaging qualities, as well as the size, shape, surface charge, and chemical structure of the nanoparticles, affect the migration process [70]. When nanoparticles get into food, they may interact with biological systems and cause negative consequences. Several studies have indicated that nanoparticles have the potential to accumulate within organs and tissues, hence resulting in toxicity [75]. Moreover, the potential impact of nanoparticles on the environment remains uncertain upon their introduction into ecological systems via food waste disposal [76].

### 5.3. Antioxidants

Antioxidants have the capability to minimize the oxidation rate and improve the stabilization of the substance through self-degradation [77]. Although these antioxidants have advantages in preserving food quality, they are concerning for their potential migration from food packaging to food. The migration occurs when antioxidants are transferred from the packaging material to the food ingredient. Multiple processes can facilitate this process, including diffusion, adsorption, or direct contact [7,17]. The degree of migration can be influenced by several factors, including temperature, time, and the specific characteristics of the food and packaging material [17]. Food packaging commonly incorporates many antioxidants, viz., butylated hydroxytoluene, butylated hydroxyanisole, tertiary butylhydroquinone polyphenols, propyl gallate, tocopherols (vitamin E), carotenoids, and ascorbic acid (vitamin C) [60]. Moreover, the chemical antioxidants typically employed in plastic packaging materials include Tinuvin 776 DF, Tinuvin 234, Tinuvin P, Tinuvin 326, Irganox 1010, Irganox168, Irganox P-EPQ, and Irganox 1330 [17]. The application of antioxidants in packaging materials, viz., plastics, coatings, and adhesives, prolong the shelf life of food products by retarding the progress of oxidation.

Food safety authorities monitor the migration of antioxidants into food to ensure that the levels of these compounds in food do not surpass safety limits [78]. Research has revealed that antioxidants can migrate from packaging materials, however, the concentrations are typically minimal and do not pose a substantial health risk under typical habits of consumption [44]. Nevertheless, there are concerns over the possible enduring effects of prolonged exposure to small quantities of antioxidants via food packaging. Several studies have indicated that some antioxidants may potentially have adverse effect on human health, including the development of cancer or disturbance of hormonal balance, particularly when exposed for extended periods [44,79,80]. To reduce the migration of antioxidants into food, food producers must carefully select packaging materials and suitably employ them. Furthermore, continuous research is being carried out to explore alternate packaging materials and technologies that can mitigate migration while ensuring food quality and safety.

### 5.4. Light Stabilizers

Light stabilizers play an important role as additives in food packaging materials since they protect the packaged food against degradation resulting from light exposure [81]. UV radiation is effectively absorbed or scattered by these items, restricting its transmission to the food and minimizing any adverse effects such as the development of off-flavors, loss of nutrients, or color changes [82]. Food packaging applications involve various light stabilizers, such as hindered amine light stabilizers (HALS), benzotriazoles, and benzophenones [83]. The selection of these compounds is based on their capability to absorb or reflect ultraviolet (UV) radiation efficiently, therefore maintaining the packed food [82]. Light stabilizers can frequently be used along with other additives to ensure complete protection against deterioration caused by light. Moreover, light stabilizers enhance the enduring weathering properties of polymers, particularly polyolefins [7]. Polymeric-hindered amines are frequently employed as light stabilizers in polyolefins. These amines are detected using advanced analytical techniques based on ultra-performance liquid chromatography, employing detectors that operate at two different wavelengths, such as UV and visible [17].

Nevertheless, some concerns occur regarding the migration of these light stabilizers into the food from the packaging that contains them if corrosion occurs [7]. Migration may be influenced by several variables, including temperature, time, and the properties of the food source [17]. To address this issue, food safety authorities enforce regulations on the migration of light stabilizers, establishing precise migration limits to ensure that the amounts of these substances in food stay below acceptable levels.

### 5.5. Thermal Stabilizers

The utilization of thermal stabilizers in food packaging materials serves the purpose of minimizing the degradation of the packaging material caused by prolonged heat exposure during the various stages of manufacturing, storage, and transportation. These chemicals have a crucial role in preserving the structural integrity of the packaging, assuring its continued functionality and preventing any potential contamination of the food it contains. Metal-based stabilizers, such as calcium, zinc, or magnesium stearates, are a common category of thermal stabilizers [84]. Stabilizers are included in polymers employed in food packaging to minimize material degradation under elevated temperatures. Under elevated temperatures, these stabilizers can go from the packaging to the food, especially in fatty or oily foods, because they strongly attract lipids [85]. Organophosphorus compounds, epoxy compounds, and beta diketones are other forms of thermal stabilizers commonly employed in plasticizers to enhance the flexibility and durability of packaging materials [43]. Epoxy stabilizers are often obtained from linseed, epoxidized soybean, and sunflower oils. These stabilizers are found in many food-packaging plastics, serving as heat stabilizers, lubricants, and plasticizers [43]. The migration of other heat stabilizers into food, particularly in acidic or fatty food items, presents a potential contamination hazard.

The possible health risks associated with exposure to thermal stabilizers have generated significant concerns. For example, many stabilizers derived from metals, like lead or cadmium compounds, possess toxic properties and have the potential to accumulate inside the human body, hence resulting in detrimental health consequences [86]. Health concerns like developmental and neurological diseases have been associated with organophosphorus chemicals [87]. Manufacturers employ barrier layers in packaging materials to minimize direct contact between thermal stabilizers and food, thereby reducing the stabilizers’ migration into the food.

### 5.6. Monomers and Oligomers

Monomers and oligomers are constituents of small molecular structures that find application in manufacturing food packaging materials, including plastics [88]. The observation of chemical migration from the packaging material to the food has generated apprehension regarding the potential ramifications for both human health and food safety. Monomers serve as the essential components of polymers, which are complex compounds arising from the repetitive interconnection of monomers [17]. Oligomers are intermediate molecules and consist of a small number of monomer units, usually ranging from 2 to 10 units.

Styrene, a frequently used monomer, is employed in the manufacturing process of PS, a material that is extensively utilized in food packaging and is in contact with food products. PS is primarily employed to contain various dairy products such as cottage cheese, ice cream, fruit juice, yogurt, other beverages, poultry, meat, bread items, and fresh vegetables [89]. According to Vodicka [90], it has been shown that a styrene monomer has the potential to undergo degradation into its corresponding oxide. This oxide has been shown to have significant mutagenesis properties and, when metabolized within the human body, can generate hippuric acid, which can be eliminated through urine. The potential consequences of exposure to styrene include organ toxicity, skin irritation, eyes and lungs, and the concurrent inhibition of central nervous system function.

Vinyl chloride monomer is a colorless gas under standard pressure and temperature conditions [91]. Nevertheless, it is commonly employed as a polyvinyl chloride (PVC) packaging and pressurized liquid within steel cylinders. The chemical is highly toxic, and its prescribed limits have been set in food packaging since the 1970s [92]. Plastic PVC bottles and food packaging have been shown to leach vinyl chloride. However, governmental bodies, such as the Food and Drug Administration (FDA), have implemented regulations on the permissible levels of vinyl chloride in food packaging materials [7].

BPA is a frequently encountered monomer employed in food packaging, primarily utilized in manufacturing polycarbonate plastics and epoxy resins [17]. BPA can be transported from packaging materials to food, particularly acidic or high-fat food products, presenting significant health hazards. BPA has been linked to a plethora of health concerns, encompassing hormone imbalances, reproductive dysfunctions, and developmental impairments [93].

Plastic Terephthalate Oligomer (PET) is extensively utilized in the manufacturing of various bottles and trays employed in the realm of food packaging. These applications include mineral water, carbonated drinks, beer, juice, vegetables, milk, and other perishable food items [94]. Due to its excellent temperature resistance, it is readily suitable for manufacturing plates for traditional thermal processing and microwaves. However, studies have shown that PET demonstrates very little low molecular weight oligomers, varying from dimers to pentamers. Acetaldehyde is the principal volatile molecule found in PET and is highly significant since it affects the olfactory features, especially in cola-type drinks [95].

Polyamides or nylons are among the rare polymers utilized for food containment in cooking. Nevertheless, surveys suggest that small amounts of residual caprolactam, nylon oligomers, and nylon monomers have the potential to disperse in boiling water [96,97]. Typically, these chemicals are non-toxic, although they can give foods a bitter taste [98]. Furthermore, it has been demonstrated that the inhalation of elevated concentrations of κ-caprolactam can result in an appearance of inflammation symptoms and the sensation of burning in the eyes, nose, throat, and skin. Additionally, individuals may experience headaches, malaise, confusion, and nerve irritation [7]. Both monomers and oligomers have the ability to get inside food through a phenomenon known as diffusion. This process is subject to several conditions, including temperature, time, food composition, and characteristics of the packaging materials [7].

### 5.7. From Printing Inks and Adhesives

Food packaging utilizes printing inks to offer labelling, branding, and decorative components. These substances consist of different constituents, such as pigments, binders, solvents, and additives, that have the possibility to get into food and cause health hazards [35]. A prevalent issue associated with printing inks is the existence of heavy metals, including lead, cadmium, and mercury, which serve as pigments or catalysts. Metals can migrate from the package to food, particularly in acidic or fatty foods, and gradually build up in the body, resulting in adverse health consequences. In food product packaging, it is common for a singular layer of material to incorporate printed inks to convey the product information to purchasers [99]. The application of such packaging for food storage could potentially elevate the likelihood of pigments or printing colors being transferred to the food, thereby presenting a risk to its quality and safety. UV-curable inks and varnishes, which may be printed, are frequently employed in packaging. They typically have three main elements: a monomer, an initiator, and a pigment [100]. During the application procedure, the photoinitiator in the ink is transformed into a free radical by exposure to a UV source. This free radical subsequently reacts with the introduced monomers, initiating polymerization. UV-cured inks may contain excessive residuals of photoinitiators or monomers due to an imbalanced formulation of the monomers and photoinitiators, as well as faulty functioning of the UV source [17]. As a result, absorption of these molecules into a food matrix would compromise the safety of the food and modify its sensory qualities. Furthermore, contamination from the migratory species’ interaction with the food might result in a reduction in its nutritional value and quality. It has been shown that benzophenone migration, a frequently used odorless photoinitiator, generates alkyl benzoates, which can lead to undesirable flavors [101]. Similarly, plasticizers, which are commonly added to printing inks and packaging materials to provide qualities like stickiness and flexibility, have the potential to migrate from the packaging sheets and contaminate food [102].

In food packaging, adhesives are frequently utilized to bind numerous components of the packaging materials. These adhesives may be based on various technologies, including hot melt, water-based, or solvent-based adhesives [103]. Although adhesives play an essential role in maintaining the functioning and integrity of food packaging, there are concerns over their ability to contaminate food and compromise food safety. The migration of components like monomers, oligomers, or residual solvents is one of the critical issues with adhesives. Adhesives may contain these chemicals in the manufacturing process or product formulations. These substances can permeate the packaging material when food comes into contact with them, contaminating the food [17]. For instance, volatile organic compounds (VOCs) included in solvent-based adhesives can evaporate and permeate into food, particularly when the adhesive is applied as a liquid and subsequently dried or cured [104]. These VOCs might contain substances with established potential health risks, such as toluene, ethyl acetate, or methyl ethyl ketone [104]. On the other hand, water is the primary solvent of water-based adhesives, which lowers the possibility of VOC migration [103]. However, they could still include additional ingredients or components, such as stabilizers, preservatives, or surfactants, that might migrate into food. Even though these substances are typically regarded as harmless at low concentrations, their influx into food is nonetheless regulated for the safety of consumers. Another popular kind of adhesive used in food packaging is hot melt adhesive, especially when a strong bond is needed. These adhesives are poured while still molten, solidifying as they cool [105]. Hot melt adhesives are usually accepted as safe for use in food packaging, nonetheless, there are concerns over potential component migration, particularly regarding processing aids or polymer additives. It is crucial to consider the packaging type and unique attributes of the food product when making adhesive selections. For example, using a hot melt is unsuitable for wrapping milk chocolate bars with adhesive. Migrants from printed packaging surfaces can also readily migrate to adhesive layers, particularly in cases where packaging is layered [106]. Consequently, during the packaging process, these migrants may eventually reach the food matrix. The likelihood of possible contact migration of migrants is considerably raised in the scenario of multilayer packaging systems, like laminates. Several polymers are layered with non-polymeric materials (such metals) to create multilayer laminates, which are intricate packaging materials with specific packaging properties. Even though adhesives are necessary for food packaging, there are concerns regarding their ability to contaminate food and affect food safety. For food packaging materials to be safe and to reduce the migration of hazardous substances, manufacturers must use suitable adhesives and comply with regulations. The mechanism of migration of different additives which has application in packaging material has been illustrated in Table 2.

## 6. Toxicity Evaluation of Migrated Compounds to Food Products

The migration of chemicals from food packaging materials into food products raises significant toxicological concerns. These migrating species encompass a wide range of substances, including residual monomers like BPA, low molecular weight additives such as plasticizers and antioxidants, oligomers like cyclic polyesters, and degradation products originating from the packaging polymers themselves [25]. Many of these compounds have been identified as potential toxicants, capable of inducing adverse effects ranging from endocrine disruption to carcinogenicity and reproductive toxicity if ingested above permissible exposure levels [127]. Numerous recent studies have quantified alarming migration levels of these compounds that exceed regulatory thresholds set by authorities like USFDA and EFSA. For example, it has been observed that phthalates, which are frequently employed as plasticizers in PVC food packaging, migrate into food simulants from cling films at concentrations greater than 30% above the regulatory limits set by the European Union [24,128]. Similarly, BPA, a well-known endocrine disruptor, has been detected migrating from polycarbonate and PET bottles into food simulants and bottled water, respectively, at levels violating safety criteria [129]. Furthermore, oxidation and hydrolysis products of PET, such as acetaldehyde and 2,4-di-tert-butylphenol, have been quantified in fruit beverages, raising additional health concerns [30]. Equally concerning are the high levels of microplastics, predominantly < 5 μm, detected migrating from common plastic containers like polypropylene, PET, and polystyrene into food simulants, significantly exacerbating their release by heating and fatty food types [130]. These findings underscore the urgency of addressing migration issues and implementing stringent regulations to protect consumer health from potential toxic exposures associated with food packaging materials.

To assess the hazardous nature of migrants from food packaging materials, rigorous toxicological assessment protocols are employed, adhering to international guidelines and regulations [131]. These protocols encompass both in vitro and in vivo tests, evaluating various endpoints such as genotoxicity, cytotoxicity, mutagenicity, reproductive toxicity, and endocrine disruption [132]. For example, studies have shown that migrant compounds from PET can induce cytotoxic effects, including DNA damage in exposed cells, suggesting potential in vivo toxicity [130]. Similarly, cyclic polyester oligomers migrating from polyurethane adhesives in multilayer packaging, which exceeded regulatory limits in the majority of samples tested (19 out of 20 samples), exhibited weak androgen receptor antagonism in vitro, though without aryl hydrocarbon receptor activity or binding to thyroid hormone transport proteins [114]. Subsequent simulated digestion studies have elucidated the bioaccessibility and metabolic transformation pathways of these oligomers [133].

Advanced analytical techniques such as the liquid chromatography-mass spectrometry (LC-MS) and the gas chromatography-mass spectrometry (GC-MS) play an important role in the meticulous identification and quantification of individual migrants from food packaging materials. These techniques enable precise exposure estimation by detecting even trace amounts of substances [18]. Meanwhile, mathematical migration models, grounded in diffusion and partition theories, complement analytical efforts by simulating various exposure scenarios considering packaging formulations and food contact conditions [134]. Despite their utility, challenges arise from the presence of multiple co-migrants and the intricate matrix effects inherent in food packaging materials, making it difficult to accurately predict cumulative toxicity outcomes. Moving forward, adopting an integrated approach is essential for ensuring the safety of packaged food products. This approach involves synergizing migration studies, toxicological screening, computational modelling, and risk assessment frameworks. Compiling information from several sources such as toxicity evaluations, analytical findings, and prediction models allows for a thorough grasp of the possible health hazards connected to packaging migration. Furthermore, the development of novel packaging materials derived from safer, inert, and ideally bio-sourced feedstocks presents an enticing opportunity to circumvent toxicity concerns linked to conventional packaging materials altogether. Embracing innovation in packaging materials not only enhances consumer safety but also contributes to sustainability efforts within the food industry.

## 7. Analytical Techniques for Monitoring Migration

Chromatographic detection is the process of identifying and quantifying analytes separated by chromatography. Chromatographic methods play an essential role in the detection and analysis of migrants from packaging materials. These methods are instrumental in separating, identifying, and quantifying various chemical compounds that may leach from packaging into food and beverages. This method aligned precursors and fragment ions using both retention and drift time, resulting in spectra that were cleaner and less complicated. Canellas et al. [135] employed a novel methodology that integrated Ion Mobility Quadrupole Time of Flight Mass Spectrometry and UHPLC to examine the migration of compounds from printed straws into a carbonated beverage. The compounds such as tris(2,4-ditert-butylphenyl)phosphate, 2-stearoylglycerol, diisodecyl adipate, 2,2,4-trimethyl-1,3-pentanediol diisobutyrate, bis(2-ethylhexyl) sebacate, bis(2-ethylbutyl)phthalate, N,N-ethylenebisstearamide, 2,3-Di(Octanoyloxy)propyl octanoate, 13,16,19-docosahexaenoic acid, methyl ester, 2,2-dimethoxy-2-phenylacetophenone, 3′,6′-bis(diethylamino)spiro[isobenzofuran-1(3H),9′-[9H]xanthene]-3-one, diphenyl (2, 4, 6-trimethylbenzoyl)-phosphine oxide, acetyl tributyl citrate, 4,4′-methylenedianiline and 2,2′-[(1-methylethylidene)bis(4,1-phenyleneoxymethylene)]bis-oxyrane were successfully identified in the range of 5 mg/kg to 60 mg/kg.

An ultra-high-pressure liquid chromatography system connected to a quadrupole time-of-flight mass spectrometer (UHPLC-qTOF-MS) was utilized by Colombo et al. [19] to study the presence and migration of numerous purposefully and unintentionally introduced PET cyclic oligomers from industrially recycled trays. Consuming 1 kg food per day can exceed the TTC limit for [TPA-EG]2 (88.9 μg/person/day), [TPA-EG]2-EG (292.5 μg/person/day), [TPA-EG]3 (109 μg/person/day), and [TPA-EG]2-EG2 (160.1 μg/person/day) for 73% of the sample. Exposure to [TPA-EG]3 is between 96.7 and 93.3 μg/person/day, while exposure to [TPA-EG]2-EG ranges from 132.3 to 182.2 μg/person/day. The data showed that when the %rPET increased, so did the concentration of all oligomers. Additionally, the outcomes demonstrated that, even under an extremely cautious exposure scenario, the exposure risk for all series of PET cyclic oligomers was comparatively low.

Using a QuEChERS clean-up protocol, Diamantidou et al. [113] devised the UHPLC-qTOF-MS method to determine the concentration of first-series cyclic PET oligomers in virgin olive oil (VOO). Following storage, the concentrations of cyclonic oligomers were measured in PET bottles, ranging from 587 to 2950 µg L^−1^, 38.8 to 198 µg L^−1^, and 0.51 to 9.32 µg L^−1^, respectively. Food simulants containing cyclic PET pentamers were identified through migration testing utilizing rPET bottles at levels of 2.14 µg L^−1^ and 2.57 µg L^−1^. The quantification of H-[TPA-EG]2-H, H-[TPA-EG]2-OH, and H-[TPA-EG]3-EG-OH in both types of PET is conducted below 115 μg L^−1^ concentration. In contrast, the concentrations of H-[TPA-EG]3-O-C_2_H_5_ and H-[TPA-EG]3-OH in recycled material and vPET, respectively, range from 378.7 to 822.4 μg L^−1^ and 2845 to 4284 μg L^−1^, respectively. These values suggest that the migration from vPET is more pronounced.

The validation study by Forero et al. [20] quantified acrolitrile monomer in polypropylene food packaging via ambient gas chromatography incorporating flame ionization. The migration of acrylonitrile from polypropylene granules was investigated in food simulant water and ethanol (50% *v*/*v*) at two temperatures (20 ± 1 °C and 44 ± 2 °C) for a duration of six weeks, which is comparable to the shelf life of a bottle. The HS-GC-FID chromatograms of the migration process in polypropylene food packaging subjecting to different degradation processes report a recovery range of 96.2% and 100.8% of acrylonitrile with a concentration of 0.10 ± 0.04 µg kg^−1^. Based on migration kinetics, the maximum concentration in the evaluated circumstances was calculated to be 16.4–16.6 µg kg^−1^, which is below the tolerated specific migration limit stated in the regulation.

In order to assess the linearity, limit of detection, limit of quantitation, accuracy, and precision of styrene levels in a sample of eighteen chilled dairy products packaged in rigid PS from various manufacturers, Guazzotti et al. [136] utilized an adapted purge and trap gas chromatography analysis technique, together with mass spectrometry and a flame ionization detector. Styrene monomer migration into food simulants was meticulously analyzed utilizing two PS materials: extruded high-impact polystyrene (HIPS) sheets and thermoformed PS yogurt containers. The threshold for detecting styrene in yogurt was 0.4 µg/kg, whereas the limit for quantification was 1.4 µg/kg. The degree of recovery for every item of food exhibited a range of 95 to 112.5%, with styrene in yogurt demonstrating the highest accuracy at 105.2%. The migration of styrene from PS pots/cups to single-serving pieces of raw cheese ranged from 42.9 to 74.5 µg/kg. In contrast, styrene levels in PS pots/cups varied from 4.5 to 13.8 µg/kg. PS Styrene migration levels during testing are comparable to, if not greater than, those documented in refrigerated food products.

Isci [107] conducted an LC-MS/MS analytical investigation in which phthalate esters (PAEs) were assessed both quantitatively and qualitatively in 48 fruit juice samples available in Turkey. The analysis utilized the multiple reaction monitoring (MRM) mode and precursor-product ion transitions. The average DEHP concentration in apple juice was 3.39 ng/mL, whereas apricot juice had the highest average (3.65 ng/mL). The peach juices exhibited the lowest mean DBP concentration (1.98 ng/mL), whereas the apricot juices exhibited the highest mean (2.45 ng/mL). The DIDP concentrations of various fruit juice brands were assessed and determined to range from 1.58 to 1.93 ng/mL, 1.89 to 2.60 ng/mL, 1.95 to 2.90 ng/mL, 2.52 to 2.92 ng/mL, 2.7 to 3.01 ng/mL, and 2.7 to 3.06 ng/mL, in that order. The DBP concentrations in various brands of fruit juice varied as follows: 1.82–2.57 ng/mL, 1.52–2.20 ng/mL, 1.92–3.02 ng/mL, 2.77–3.18 ng/mL, 2.10–2.64 ng/mL, and 1.05–2.73 ng/mL, in that order. The investigated brands exhibited DINP concentrations ranging from 28.51 to 29.26 ng/mL, 25.76 to 26.15 ng/mL, 28.76 to 29.61 ng/mL, and 30.70 to 31.19 ng/mL, in that order. Several fruit juice brands were determined to contain DNOP concentrations within the following ranges: 0.42–4.68 ng/mL, 1.44–2.02 ng/mL, 1.81–2.85 ng/mL, 3.00–3.75 ng/mL, 3.48–4.13 ng/mL, and 3.41–4.46 ng/mL, respectively. In general, the findings indicate the presence of PAEs in the fruit juice samples, albeit at concentrations below the SMLs established by the EU Regulation for each substance examined: 30 mg/kg for BBP, 1.5 mg/kg for DEHP, 0.3 mg/kg for DBP, and 9 mg/kg for DINP and DIDP. Significantly, it was ascertained that the concentrations of PAE in each sample were below the specific migration limit (SML) of 1.5 mg/kg, which is the minimum threshold required by Commission Regulation (EU) No. 10/2011.

An UHPLC system, combined with a triple-quadrupole mass spectrometer, was used by Kim et al. [111] to detect eleven substances (four antioxidant degradation products, four antioxidants, three other degradation products, and contaminants) that contaminate food contact materials and migrate into food [111]. The detection limits for the eleven target compounds ranged from 0.49 to 15.63 µg/L. The detection limits for the eleven target compounds ranged from 0.49 to 15.63 µg/L. Triphenylphosphine oxide had the lowest detection limit (0.49 µg/L), whereas the detection limits for the remaining eleven compounds ranged from 1.95 to 15.63 µg/L. All four antioxidants were discovered in PP—2841 μg/L, PE—10,277 μg/L, PET—97 μg/L, PS—18 μg/L, ABS—3876 μg/L, MLF—3414 μg/L, and PVC—3552 μg/L. A non-targeted analysis of the samples unveiled 22 compounds, comprising 11 distinct components. 1,3-di-tert-butylbenzene (G4), which was identified in seven products, constituted the maximum proportion of antioxidant degradation products. Additionally, in a total of 32 instances, 2,4-di-tert-butylphenol and ten antioxidant degradation products were identified in PP, PCT, and MF; this represents the highest ratio (31.25%). At 22.30%, PP entailed the greatest risk, followed by PE at 16.20%. At 54.78%, (z)-9-octadecenamide exhibited the second-highest cumulative risk, after PP at 12.48% and PE at 27.78%. 7,9-di-tert-butyl-1-oxaspiro (4,5) deca-6,9-diene-2,8-dione exhibited the second-highest cumulative risk, amounting to 34.81%.

Utilizing Liquid Chromatography Coupled with Tandem Mass Spectrometry, Lestido-Cardama et al. [126] examined the migration of dihydroxyalkylamines and possible contaminants from packaging into food items (orange juice, gouda cheese, alcoholic beverages, surimi, and coffee capsules). N, N-bis(2-hydroxyethyl) octadecylamine was the tertiary amine commonly identified in the samples analyzed, followed by N, N-bis(2-hydroxyethyl) hexadecylamine. The detection of primary amines was limited to PEG-2 hydrogenated tallow amine. With the exception of the secondary amines 2-(hexadecylamino) ethanol and 2-(octadecylamino) ethanol, which exhibited elevated toxicity, all identified compounds belonged to class I. Seven of the analyzed packaging materials contained 2-octadecylamino-ethanol, which was also present in the PEG-2 hydrogenated tallow amine. Both target and non-target methods were implemented. Alkyl amines of the N, N-bis(2-hydroxyethyl) variety, denoted as C12, C13, C14, C15, C16, C17, and C18, were detected in an extensive majority of the examined samples. Furthermore, it was observed that several samples contained 2-(octadecylamino) ethanol, a compound not explicitly mentioned in Regulation 10/2011 and classified as highly toxic (class III) per Cramer guidelines.

The study by Sapozhnikova and Nuñez [137] involved GC-Orbitrap mass spectrometry to identify compounds migrating from paper-based food packaging materials (butcher paper, liquid egg containers, pizza boxes, and pizza box liners). Tetradecamethyl-cycloheptasiloxane, diphenyl sulfone, dibutyl phthalate, methyl dehydroabietate, and vanillin were found in much higher concentrations in pizza box liners. Diethyl phthalate levels were substantially higher in egg containers. This study found that dibutyl phthalate and benzophenone have specific migration limits of 0.6 and 0.3 mg kg^−1^, respectively, for plastic materials used in food contact. Pizza boxes contained the highest quantities of dibutyl phthalate and benzophenone (189 and 294 μg kg^−1^, respectively). These values were lower than the SML regulatory values.

Sonego et al. [138] conducted a migratory analytical investigation on perfluoroalkylated substances (PFASs) and organophosphate esters (OPEs) from food contact materials (baking paper and aluminium foil) using HPLC-ESI-MS/MS with multiple reaction monitoring. The results demonstrate that baking paper releases the most pollutants, with values ranging from 78.30 to 413.21 ng/dm^2^ for OPEs and 1.43 to 13.87 ng/dm^2^ for PFASs. Triethyl phosphate and Tris (2-butoxyethyl) phosphate are the OPEs discovered in practically all samples, with values as high as 327.37 ng/dm^2^ and 83.21 ng/dm^2^, respectively. This finding indicates that consumers are more exposed to pollutants when they consume aqueous-based acidic foods versus fatty foods for the two food contact materials under consideration.

The purpose of the study by Tan et al. [129] was to use HPLC analysis to investigate how the BPA levels in brined white cheese changed after cold storage in polyethylene terephthalate containers. BPA levels in discarded PET containers ranged from 0.000 to 0.040 μg/kg, while control group cheeses contained 0.032 to 0.094 μg/kg. BPA levels in the control group ranged from 0.443 to 0.747 μg/L in brine solution and 0.927 to 1.237 μg/kg in cheese samples. The BPA concentration in the brine solution of the BPA group samples ranged from 1387 to 1894 μg/L, while the cheese samples ranged from 3702 to 4205 μg/kg. Fluctuations were noticed throughout the storage period.

Ubeda et al. [116] investigated the migration and digestion assays of cyclic polyester oligomers from food packaging adhesives. The concentration of cyclic esters in migration varied greatly, with AA-DEG migration values ranging from 20 to 994 ng g^−1^ being higher than AA-DEG-IPA-DEG values of 4 to 346 ng g^−1^. The migration of cyclic esters in multilayer materials surpassed the EU/10/2011 limit of 10 ng g^−1^ for unlisted chemicals. The digestion assay results were evaluated using UPLC-QqQ and UPLC-QTOF. The results showed that digestion reduced the concentration of the cyclic polyester AA-DEG by 43% and AA-DEG-IPA-DEG by 53.2%. In addition, AA-DEG migrated significantly more than AA-DEG-IPA-DEG, possibly as a result of its more compact structure and absence of an aromatic ring, as indicated by the data.

According to Vázquez-Loureiro et al. [139], Bisphenols and BADGEs were identified and quantified using HPLC-FLD and LC-MS/MS methods. The LC-ESI-TOF technique was employed in order to ascertain the composition of the oligomers extracted from polyester coatings. An additional three oligomers, which had been provisionally detected in the polyester coatings, were identified in a variety of food samples, including 2 TPA + BD/MBO + DEG(C), TPA + PG + EG(L), and 2 TPA + EG(L). Bisphenols and BADGE derivatives showed recoveries ranging from 63% to 102%. Carboxylic acid recoveries were between 92% and 137%. The concentrations of BADGE.2 H_2_O and BADGE.H_2_O.HCl ranged from 0.65 to 0.09 μg/g, respectively. Notwithstanding the potential adverse consequences of BPA, the concentrations identified in multiple samples surpassed the regulatory threshold. Cycloid BADGE was also higher in the majority of the examined samples than the BfR’s permitted migration threshold of 0.05 µg/g.

## 8. Regulations and Legislation in Migration

The migration of chemicals and additives from packaging materials into food is a substantial concern for public health and food safety. By establishing guidelines for the materials used in food packaging and verifying their migration potential, a variety of regulations and legislations are designed to mitigate these risks.

The USFDA regulates the safety of food packaging materials, augmented by guidelines from the Environmental Protection Agency (EPA) and the U.S. Department of Agriculture (USDA). The FDA oversees food contact materials (FCMs) under Title 21 of the Code of Federal Regulations (CFR), Parts 174–179 (Table 3). Essential elements encompass compounds that may migrate into food, the Food Additive Petition (FAP) for foreign/new substances, and the threshold of regulation (TOR) exemptions for substances that migrate in minimal quantities and provide no safety hazard. Certain materials and substances may be designated as Generally Recognized as Safe (GRAS), exempting them from premarket review by the FDA. The Food Contact Notification (FCN) process enables industries to inform the FDA about new food contact compounds and no objections from the FDA within 120 days allowing the material to be used in food packaging. The FDA controls food packaging waste and its environmental impact, requiring manufacturers to do migration tests to quantify the compounds that may transfer from packaging to food under designated conditions [140].

In Asian countries, FCMs are regulated by multiple authorities, such as China’s National Health Commission and the State Administration for Market Regulation. China’s rules encompass a range of products, including plastics, rubber, coatings, metals, glass, paper, and adhesives. The regulatory structure in Japan is primarily overseen by the Ministry of Health, Labour, and Welfare, which establishes food safety regulations. South Korea oversees FCMs via the Ministry of Food and Drug Safety encompassing a broad spectrum of materials. The Food Safety and Standards Authority of India governs FCMs under the Food Safety and Standards Act of 2006. The Thai Food and Drug Administration, under the Ministry of Public Health, regulates FCM in Thailand. FCMs in Indonesia are regulated by the National Agency of Drug and Food Control. The Ministry of Health oversees Malaysia’s food contact material laws, which are governed by the Food Act 1983 and the Food Laws 1985. The Singapore Food Agency regulation ensures adherence to local and international food safety standards [141].

Comprehensive assessments of chemical migration are mandated by the European Union’s stringent rules and regulations regarding FCM. The European Union (EU) Framework Regulation regulates FCMs at the union level, including packaging, machinery, and cooking appliances, while various directives and laws were established at the community level a long time ago. The EU Framework Regulation establishes specific requirements for the manufacture and marketing of plastic materials and articles that are intended to come into contact with food, or already in contact with food, or which can reasonably be expected to come into contact with food by conducting migration testing [140]. Specific standard analysing protocols for specific compounds have been employed for migration testing to obtain a Specific Migration Limit, which is generally characterized by the quantity of a substance that may be present in the packaging material and the quantity that could potentially migrate to the food [7]. The control of packaging materials compliance with EC regulations is a complex subject matter and the Framework Regulation has been established to fulfil the usage of the materials and articles that come in contact with food products (Table 3). Among the several challenges highlighted are the enormous number of compounds on positive lists, the lack of information on potential migrants, the lack of standardized analytical methods, the length of analytical procedures, and the practical problems in carrying them out [142]. The safety and awareness of food packaging can be further improved by educating consumers on potential hazards and safe handling practices.

**Table 3:** Global regulations and legislations for food contact materials.

Directives	Subject	Material	Reference
European Union—The European Parliament and Council			
(EC) NO 1935/2004	Framework key regulation concerning materials and articles intended to come into contact with food	All materials	[13]
2023/2006/EC	Good Manufacturing practices applicable to all food contact materials	All materials	
93/11/EEC	Release of the N-nitrosamines and N-nitrosatable substances from food contact materials used for infants.	Nitrosamines	
82/711/EEC	Regulation for migration testing of nitrosamines.		
93/8/EEC	1st amendment		
97/48/EC	2nd amendment		
2005/72/EC	Commission Directive that serves as an amendment to Directive 2002/72/EC on plastic materials and articles intended (monomers) to come into contact with food.	Monomer additives	
2004/1/EC	Amendment to Directive 96/77/EC lists specific purity criteria for food additives other than colors and sweeteners.		
2004/19/EC	Amendment to Directive 2002/72/EC updated provisions concerning the use of plasticizers		
2005/79/EC	3rd amendment		
2007/19/EC	4th amendment		
2008/39/EC	5th amendment		
(EC) 975/2009	6th amendment		
78/142/EEC	Limitation of vinyl chloride monomer in materials and articles intended to come into contact with food.	Elastomers and rubbers	
80/766/EEC	Concerning the analysis of vinyl chloride released by materials and articles intended to come into contact with food		
81/432/EEC	Relating to methods of analysis for ensuring the uniformity of testing for vinyl chloride monomer		
372/2007/EC	Authorization of transitional migration limits for plasticizers in gaskets in lids		
(EC) No 282/2008	Specific requirements for the safety of recycled plastics, including processes for decontamination and testing methods to ensure that recycled plastics are used in food contact materials.	Recycled plastic	
1895/2005/EC	Addresses the use of specific substances known as BADGE (Bisphenol A diglycidyl ether), BFDGE (Bisphenol F diglycidyl ether), and NOGE (Novolac glycidyl ether) in food contact materials	Coatings	
(EC) No 450/2009	Establishes specific rules and requirements for materials that can actively alter the condition of the food or the environment surrounding	Active and intelligent materials	
84/500/EEC	Directive applies to ceramic materials and articles that are intended to come into contact with food and beverage	Ceramics	
2005/31/EC	1st amendment		
2007/42/EC	Relating to materials and articles made of regenerated cellulose film intended to come into contact with foodstuffs.	Regenerated cellulose film	
2011/8/EU	Plastic materials and articles intended to come into contact with foodstuffs restricting the use of Bisphenol A in polycarbonate infant feeding bottles	Bisphenol A	
(EU) 2023/1627	The use of the substance bis(2-ethylhexyl) cyclohexane-1,4-dicarboxylate, as an additive (plasticizer) in poly (vinyl chloride) at up to 25% w/w in contact with aqueous, acidic, and low-alcohol foods for long-term storage at room temperature or below (refrigerated and frozen)		
(EU) No 202/2014	Favorable scientific evaluations for two additional substances, namely 2-phenyl-3,3-bis(4-hydroxyphenyl)phthalimidine (3) and 1,3-bis(isocyanatomethyl)benzene (4) added to the Union list of authorized substances as food contact material (FCM) substances		
USA—Food and Drug Administration			
21 CFR Part 174	Establishes general principles that apply to indirect food additives	All materials	[143]
21 CFR Part 175	The use of adhesives and coating components in food contact materials	All materials	
21 CFR Part 176	The use of binders, fillers, and coating materials.	Paper and paperboard	
21 CFR Part 177	Includes guidelines for using a wide range of polymers such as polyethylene, polystyrene, polycarbonate, polyvinyl chloride, etc.	Polymers	
21 CFR Part 178	Guidelines for establishing prescribed safety limits in using plasticizers, lubricants, and antimicrobial agents in food contact materials	Indirect additive	
21 CFR Part 179	Governs the production, processing, and handling of irradiation in packaging	Irradiation	
21 CFR Part 179.39	Establishes a method for assessing if a food-contact material may be excluded from classification as a food additive, provided its application leads to no migration or little migration into food	All materials	
21 CFR Part 182	Provides a list of all ingredients to be used in food contact material that are generally recognized as safe	All materials	
India—The Food Safety and Standards Authority of India			
Food Safety and Standards (Packaging) Regulations, 2018 Food Safety and Standards (Packaging and Labelling) Regulations, 2011	Framework key regulation concerning materials and articles intended to come into contact with food	All materials	[144]
China—National Health Commission (NHC) and Ministry of Health			
GB 4806 Series	Establishes regulations for GB materials such as plastics, rubber, coatings, metal, glass, paper, and adhesives that come into contact with food.	All materials	[145]
Thailand—Thai Food and Drug Administration			
Food Act B.E. 2522 (1979)	Framework key regulation concerning materials and articles intended to come into contact with food	All materials	[146]
Indonesia—National Agency of Drug and Food Control			
BPOM Regulation No. HK.03.1.23.07.11.6664	Establishes rules for the use of packaging materials in food contact, including safety assessments and migration limits.	All materials	[147]
Australia—Food Standards Australia New Zealand			
AS 2070-1999	Establishes usage of plastics in food contact applications	Plastics	[148]
Standard 1.4.3	Establishes the specific limits for chemical migrants from FCM	All materials	
National Environment Protection Measure (2011)	Provides guidelines for the use of recycled materials in food contact materials	Recycled materials	
Canada—Canadian Food Inspection Agency			
CAN/BNQ-0017-088	Framework key regulation concerning materials and articles intended to come into contact with food	Bioplastics	[149]
UK—Department for Environment Food and Rural Affairs			
Food Safety Act 1990	Framework key regulation concerning materials and articles intended to come into contact with food	All materials	[150]
The Materials and Articles in Contact with Food (England) Regulations 2012	Framework for Implementation of EU regulations on FCM.	All materials	
UK REACH	Provides guidelines and list of chemicals to be used in FCM	All materials	

## 9. Conclusions and Outlook

The migration of chemical compounds from packaging materials into the food system is a complicated and critical issue, presenting substantial health and safety problems that need the attention of various stakeholders, including researchers, industry professionals, and regulatory authorities. Challenges remain in completely comprehending migration pathways and efficiently minimizing exposure to hazardous compounds. This review examined essential mechanisms including contact migration, gas phase migration, penetration migration, set-off migration etc., along with variables affecting these processes such as temperature, food composition, and storage conditions. Comprehending these interactions is essential for enhancing food packaging safety. Advanced analytical methods are necessary to precisely monitor migrating chemicals and guarantee adherence to regulatory standards. Furthermore, research need to concentrate on developing sophisticated packaging materials that reduce chemical migration while maintaining food quality. Innovations like bio-based packaging and improved barrier coatings provide viable alternatives. Moreover, advanced analytical methods, such as real-time monitoring, might increase the detection of even minute quantities of hazardous substances. Regulatory guidelines are crucial for establishing migration limitations, nevertheless, continuous study is vital to refine these standards and design more effective mitigation techniques.

## Figures and Tables

**Table 1:** Migration of chemicals/additives from food packaging material to food system in storage and heating conditions.

Food Products	Packaging Materials	Migration Compounds	Storage Conditions	Heating Conditions	Observations	References
Water	PVDC	DEHP	-	25, 60, and 80 °C	The migration of Phthalate was enhanced in PVDC/Phthalate/food simulant (water) systems by high contact energy and fractional free volume.	[44]
Milk powder	Aluminium Can	BPA, caprolactam	25 °C for 6 months	60, 150, and 250 °C	Milk powder showed a low migration rate of caprolactam and BPA	[45]
Tunny bonito	PP	DEHP	4 °C for 4 months	-	Longer periods of contact with the packaging material resulted in higher migration. The higher lipid content of food packed in PP inhibited the migration of DEHP into food.	[46]
Bean, meat, cakes, potato, milk, and aquatic products	Glass containers	DEHP	−4.3 to 25.1 °C for 1 to 150 days	-	Temperature and time both accelerated the DEHP migration into food. Compared to older adults, infants and youngsters are more likely to suffer adverse effects from DEHP.	[47]
Parboiled rice	Plastic bag	BPA, caprolactam	25 °C for 6 months	60, 150, and 250 °C	Rice did not contain caprolactam. However, migration of BPA occurred during storage.	[45]
Hot tea and milk	PS	Monomer styrene	4 °C for 7 days	20, 60, and 100 °C for 10, 30, and 60 min, respectively	The study’s findings showed that the migration of styrene monomer from polystyrene disposable into hot drinks depended on the beverages’ temperature and fat content.	[10]
Cereal	Aluminum foil	BPA, caprolactam	25 °C for 6 months	60, 150, and 250 °C	Cereals did not contain caprolactam, however, migration of BPA occurred during storage	[45]
Ethanol/water and coconut oil	PET	Tinuvin	Up to 70 days at room temperature	40–70 °C	Very little migration will occur because of the extremely low diffusion of UV stabilizers from PET. Studies conducted at 40 °C, which replicated prolonged room temperature usage, indicated that quite a few compounds would be transferred to food.	[48]
Ethanol simulant	Baking paper, microwave tray, plate, and cup	OPEs	-	1 h, 2 h, and 10 days at 40, 70, and 100 °C, respectively	Utilizing food simulants, the results showed that organophosphate esters move toward foods with hydrophilic and lipophilic characteristics	[49]
Ethanol, acetic acid, and isooctane food simulant	PE, HDPE, PP, and PET	OPEs	40 °C for 10 days	-	OPEs migrated more effectively into isooctane, suggesting that plastic packaging containing OPEs may present the highest risk of exposure when used for fatty food.	[50]
Jelly powders, breadcrumbs, flour, and mashed potato	PP, PET, PLA, cellulose acetate, offset paper, and cardboard	Acrylic, vinyl, and hotmelt	40 °C for 10 days	-	Migration of these chemical compounds was found below the legal limit.	[51]
Milk, meat, and isooctane simulant	Plastic laminate films	Polyurethane	-	20, 40, and 70 °C for 10 days, 10 days, and 2 h, respectively	The migration of chemicals in laminated films is affected by temperature, time, cross-section, and nanomaterials; moreover, temperature has a significant effect on migration	[52]
Ethanol and acidic acid simulant	LDPE and PP	Polyhydric alcohols, diethylene glycol, dibasic acids, adipic, and phthalic/isophthalic	-	121 °C for 2 h	The findings of this study on cyclic oligoesters, including their molecular makeup, range of migration concentrations, and frequency of occurrence, may be beneficial for assessing regulatory risks in the future.	[53]
Water, acetic acid, and ethanol simulant	PET, PE, PP, and PS	Pb, Cd, Cr, Hg, and Sb	-	70 °C for 2 h	The migration of Pb, Cd, Hg, and Cr from all the samples is within the EU Regulation’s permitted level.	[54]
Citric acid simulant	PET	Cd, Cr, Pb, and Sb	7.2 to 22.2 °C for 1, 7 or 14 days	-	The migration of these metals for PET is not a concern for fresh fruit and vegetables.	[55]
Ethanol simulant	PP	TiO_2_ nanoparticles	180 days at room temperature	50 °C for 7 and 10 days	Negligible migration of TiO_2_ in dairy products	[56]
Acetic acid and ethanol simulant	LDPE	Nano clay	-	40 and 70 °C for 10 days and 2 h, respectively	The migrated amount of nanoparticles is very low	[57]
Bread, butter, milk powder, orange juice, and fresh carrot	PE	Nanosilver	7 and 10 days at 40 °C	-	Migration evaluation has found food samples containing negligible amounts of nano-silver particles.	[58]
Cola, orange juice, and rice wine	PP and PE	BHA, BHT, and TBHQ	10, 25, and 40 °C for 5 days	-	Temperature showed an insignificant effect on antioxidant migration	[59]
Water, acetic acid, and ethanol simulant	PP	BHA, BHT, and TBHQ	40 °C for 10 days	-	Among the crucial attributes of the fatty food packaging films, antioxidants demonstrated improved mechanical performance, attractiveness, and potent antioxidant activity.	[60]
Ethanol simulant	PP	BHA and BHT	40 °C for 10 days	-	Processing conditions had a severe impact on antioxidant migration	[61]

Polyethylene (PE), polypropylene (PP), butylated hydroxyanisole (BHA), butylated hydroxytoluene (BHT), tert-butylhydroquinone (TBHQ), polyethylene terephthalate (PET), high-density polyethylene (HDPE), low-density polyethylene (LDPE), and polystyrene (PS), polyvinylidene chloride (PVDC), polylactic acid (PLA), organophosphate esters (OPEs), phthalate (DEHP), BPA, butylated hydroxyanisole (BHA), butylated hydroxytoluene (BHT), tert-butylhydroquinone (TBHQ), cadmium (Cd), mercury (Hg), antimony (Sb), lead (Pb), and chromium (Cr).

**Table 2:** Migration mechanism of different additives and toxicity associated in the application.

Additives	Chemical Compound	Application	Mechanism	Toxicity	Reference
Plasticizers	Phthalates and adipic acid esters	Production of cap-sealing polymers, sealing gaskets for food containers, PVC films	Characterized by low molecular weight, facilitating the package-to-food migration.	Among various phthalates di-2-ethylhexyl phthalate is found to be toxic and causes hormonal imbalances, increasing the risk of cancer, and developmental and reproductive toxicity.	[107]
Slip additives	Fatty acid amides—palmitamide, stearyl amide, stearamide, oleamide and erucamide	Used in Polyolefins, Poly Styrene, Poly Vinyl Chloride for lubrication properties	Migration from packaging films to food lipids causes rancidity and off-odors.	Imparts changes in taste, odor, or texture. However, the impact is typically minimal and within acceptable limits.	[10,108,109]
Light stabilizers	Polymeric hindered amines	Enduring weather resistance	The molecular size, polarity, permeability, affinity, and solubility properties of light stabilizers in the packaging material can affect migration rates.	Some individuals may develop skin sensitivities or allergies upon direct contact with certain light stabilizers	[33,110]
Antioxidants	Tocopherols, tocotrienols, ascorbate, vitamin A, carotenoids, Selenium, phytochemicals, Irgafos 168, Irganox 1076, Irganox 1330, Irganox 1010, Irganox 1035, Cyanox 2246, butylated hydroxytoluene (BHT), 2-3-*t*-butyl-4-hydroxyanisole (BHA)	To develop active packaging for food products considering the necessity to reduce health risks related to preservatives and additives.	Excessive amounts of antioxidants undergo rapid degradation resulting in a significant loss and thus much-reduced levels of active compounds for food protection at a later time	Excessive intake of antioxidants may lead to adverse effects, including nausea, diarrhea, gastrointestinal disturbances, anticoagulants, kidney stones, risk of lung cancer, hair loss, and leading to nutritional deficiency.	[111]
Printing Inks and Adhesives	Hydrocarbons, alcohols, glycol ethers, ketones and esters	Used in the form of dissolutions or dispersions in solvents	These are organic substances composed of hydrocarbons, alcohols, glycol ethers, ketones and esters, which may present migration into foods by directly contacting them or through the free space in the interior of the package	Toxic components in printing inks may include skin irritation, eye irritation, respiratory issues and neurological disorder.	[27,33,108,112]
Monomers and Oligomers	Styrene, Isocyanates, Vinyl chloride, Acrylonitrile, Polyethylene terephthalate, Caprolactam/polyamides	Used in dairy food cartons, meat trays, cookie trays, egg cartons, and drinking cups. In the manufacturing of polyurethane polymers and adhesive materials. Used as a liquid under pressure in steel cylinders and is applied in PVC packages. Applied as a starting material for the formation of synthetic fibres, resins, plastics, elastomers and rubbers. Thermoplastic polyester, which is produced through condensation polymerisation after performing an esterification reaction with the use of monoethylene glycol and terephthalic acid or dimethyl terephthalate. Also known as nylon used in contact with foods during thermal processing.	Degraded to styrene oxide and produces hippuric acid. A limited number of 12 isocyanates are accepted for applications in food packages and must range below 1.0 mg kg^−1^ Leach from plastic PVC bottles or food packages and leach from plastic PVC bottles or food packages. Undesirable reduction in the shelf life of liquid foods packaged in bottles made of materials such as polypropylene. Migration is mostly due to elevated temperature and long-term storage. Antimony trioxide (Sb_2_O_3_) is used as a catalyst in the polycondensation stage for the manufacture of bottle-grade PET resin and is unable to recover 100% of the catalyst, therefore a residual of Sb is retained in the resin and can migrate to food products.	The most commonly presented toxic effects due to exposure are irritation of the skin, eyes and respiratory tract and depression of the central nervous system and increased risk of cancer.	[27,113,114,115,116]
Metal	(1) Tin (2) Lead (3) Aluminium (4) Chromium	(1) Canned foods and beverages in unlacquered or partially lacquered tinplate cans. (2) Used in metal packages. (3) Used in cooking utensils, storage containers, and packages. (4) Passivation is a commonly used Cr treatment to reduce the susceptibility of oxidation and enhance the adhesion of enamel.	The major reason for the interaction of food product and metal packaging occurs mainly through these discontinuities in lacquering, which can be dissolved in the food. This affects the sensory properties and shelf life the food packed in metal containers.	Promotes severe health issues such as acute gastrointestinal disorders, central nervous system disorder, carcinogenic, mutagenic, dialysis encephalopathy, osteodystrophy, and microcytic anemia.	[117,118,119]
Coatings	Epoxy resins—Bisphenol A diglycidyl ethers (BADGE)	Used for coating internally the surfaces of two-piece DRD food cans and as starting materials for cold-cured epoxy resins.	Migration of unreacted epoxy groups of BADGE is mostly due to UV degradation of plastics.	Abnormality in liver function, hematopoietic dysfunction, fatigue, gastric pain and weakness.	[120]
Paper	(1) Dioxins (2) Benzophenone (3) Nitrosamines (4) Chlorophenols and chloroanisoles	(1) Polychlorinated dibenzo-*p*-dioxins and polychlorinated dibenzofurans are used for manufacturing paper food packages. (2) Used as photo initiator for inks and wetting agent for pigments. (3) Used in papers and waxed containers. (4) Applied to produce fungicides, biocides and herbicide intermediates.	Polychlorinated dibenzo-*p*-dioxins and polychlorinated dibenzofurans migration majorly occur through the length of storage, the fat content of the food product, and improper curing of dioxins. Since only a limited amount of the initiator is used up during UV treatment for printing ink adsorption, it is not completely removed from the printing material and migration can occur through the open structure of packaging material. Migration of nitrosamines mostly happens under high-temperature environments, acidic levels of food, and the duration of contact between food and package. Migration of Chlorophenols and chloroanisoles involves fat content of food, pH of food, presence of antioxidants, recycled packaging materials, storage temperature, and contact time.	Carcinogenic, chronic heart disease, cerebrovascular disease, endocrine disruptor, thymic atrophy, developmental disorder during pregnancy, hypercholesterolemia, hyperlipemia, hirsutism, and necrotic lesions	[112,121,122,123,124]
Other Contaminants	(1) Diphenyl thiourea (2) Hydrogen peroxide (3) Lubricant	(1) Thermal stabilizer (2) Processing agent (3) Off-flavor in two-piece cans	The contamination of food is due to other factors that are unintentionally added or occur in food due to wrong processing practices.	Chronic exposure can lead to serious health issues, particularly in the nervous system, kidneys, and reproductive system.	[108,125,126]

## Data Availability

No new data were created or analyzed in this study. Data sharing is not applicable to this article.
